# Prenatal diagnosis of fetal microhydranencephaly: a case report and literature review

**DOI:** 10.1186/s12884-020-03400-1

**Published:** 2020-11-11

**Authors:** Takahiro Omoto, Toshifumi Takahashi, Keiya Fujimori, Shogo Kin

**Affiliations:** 1 Department of Obstetrics and Gynecology, Takeda General Hospital, 965- 8585 Aidu Wakamatsu, Japan; 2 Department of Obstetrics and Gynecology, Fukushima Medical University School of Medicine, 1 Hikarigaoka, 960-1295 Fukushima City, Japan; 3 Fukushima Medical Center for Children and Women, Fukushima Medical University, 1 Hikarigaoka, 960-1295 Fukushima City, Japan

**Keywords:** Microhydranencephaly, Anencephaly, Prenatal diagnosis, Microcephalus, Hydrocephalus

## Abstract

**Background:**

The prenatal diagnosis of microhydranencephaly is important and needs to be distinguished from anencephaly, because unlike anencephaly, fetuses with microhydranencephaly can survive after birth. Herein, we report a case of microhydranencephaly that was diagnosed and distinguished from anencephaly prenatally.

**Case presentation:**

The patient was an 18-year-old woman, 2 gravida nullipara, who presented at 15 weeks of gestation. Ultrasonography showed a normal biparietal diameter (BPD) and no major anomalies. At 23 weeks of gestation, an ultrasound examination revealed a BPD of 40 mm (-5.3 standard deviation, SD). At 29 weeks, anencephaly was suspected despite difficulty in visually examining the fetal head above the orbit. At 34 weeks, insertion of a metreurynter made it possible to observe the skull. Three-dimensional computed tomography (CT) and magnetic resonance imaging (MRI) confirmed the presence of the fetal skull, a prominent occipital bone, sloping forehead, marked microcephaly, cerebral loss, and excess cerebrospinal fluid. This allowed differentiation between microhydranencephaly and anencephaly. She delivered vaginally at 37 weeks, and the child had a birth weight of 2342 g and a head circumference of 24 cm (-5.4 SD). The baby’s head was flat above the forehead, with a suspected partial head defect. The baby received desmopressin acetate due to central diabetes insipidus 6 months after birth.

**Conclusions:**

The use of multiple imaging modalities and physical manipulation of the fetal head are required to accurately differentiate between microhydranencephaly and anencephaly.

## Background

Microhydranencephaly is a malformation in which all or most of the cerebral hemispheres are replaced by membranous structures filled with cerebrospinal fluid, accompanied by microcephaly [1–3]. Microhydranencephaly manifests as severe mental retardation and easy spasticity due to the hypoplastic brain. It is distinguished from anencephaly by the presence of a normal skull and meninges [3, 4]. However, unlike anencephaly, patients with microhydranencephaly can survive with severe disability; therefore, prenatal diagnosis is important for delivery and postnatal management [5]. However, there have been few reports regarding prenatal diagnosis of microhydranencephaly [3, 6]. We report a case of prenatally diagnosed microhydranencephaly, which was difficult to distinguish from anencephaly, and reviewed the current literature.

## Case presentation

**Fig. 1:**
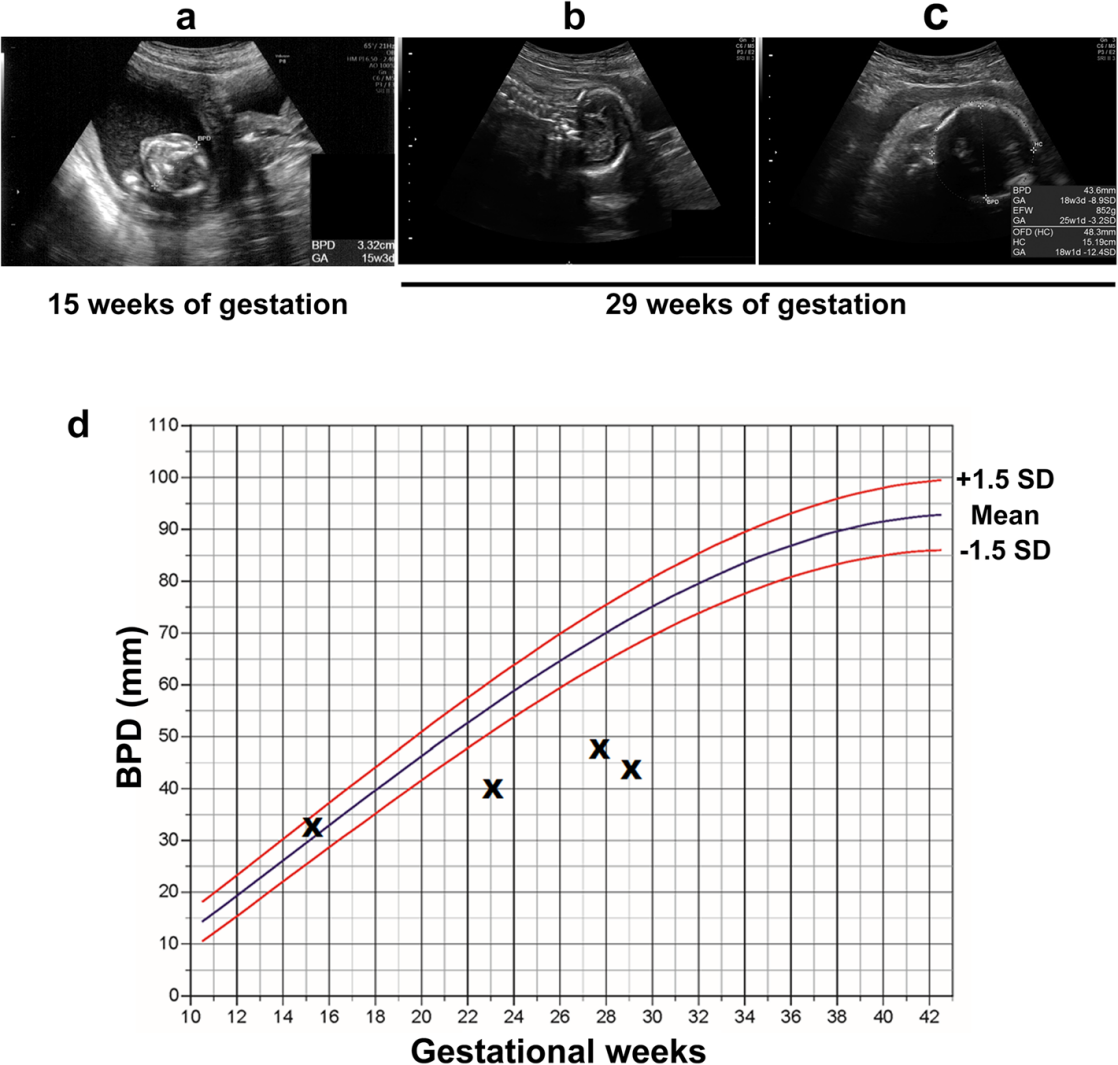
Prenatal fetal ultrasonographic findings. **a** Image of the fetal head at 15 weeks and 3 days of gestation. **b** Sagittal and **c** transverse images of the fetus at 29 weeks and 0 days of gestation. **d** A BPD growth chart. The magnitude of BPD measured by ultrasound was plotted using the x-labels. BPD, biparietal diameter

**Fig. 2:**
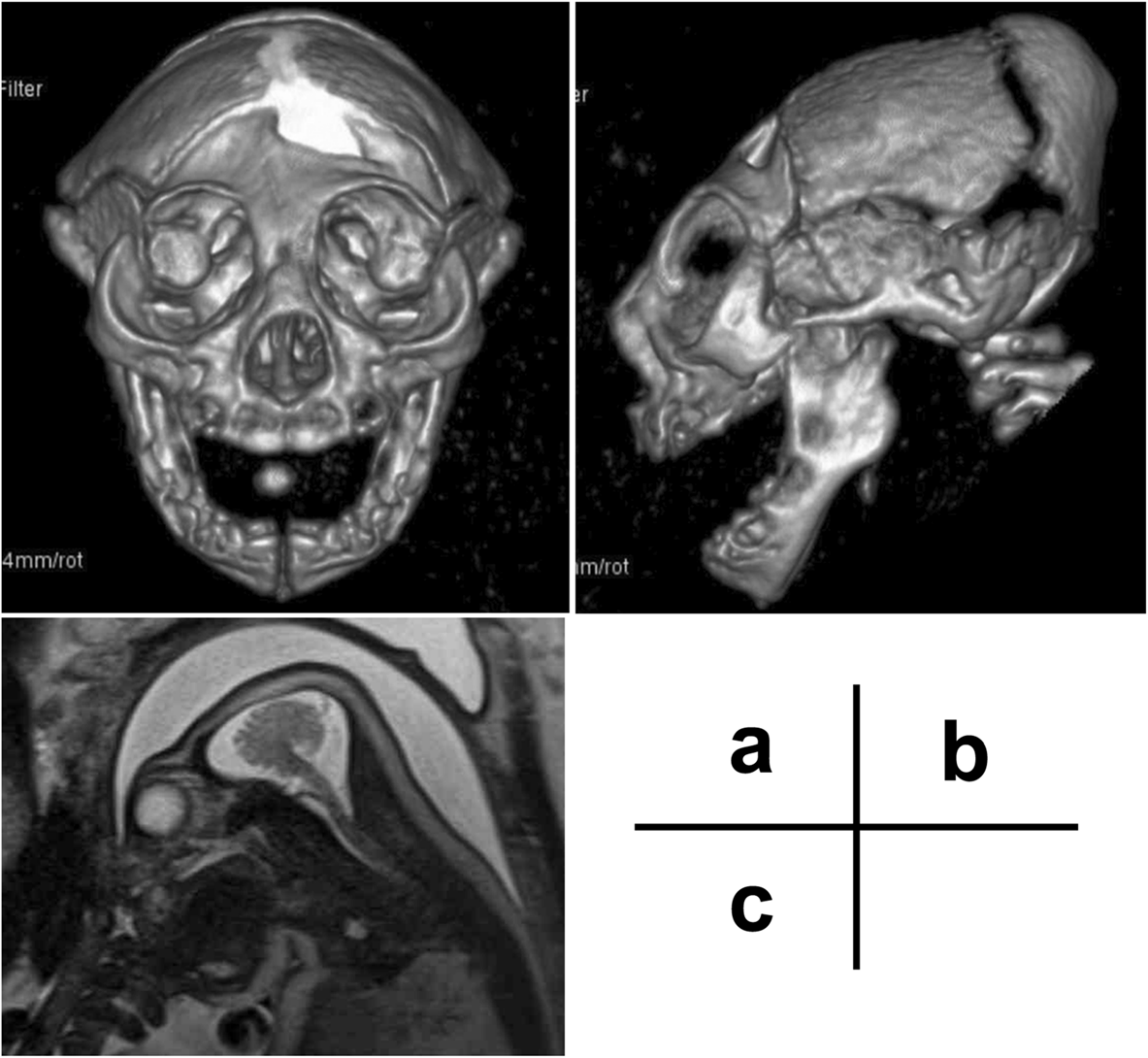
Prenatal fetal 3-dimensional computed tomography (3D-CT) and magnetic resonance imaging (MRI) scans. **a** and **b** 3D-CT images at 34 weeks of gestation showing the presence of a fetal skull, a prominent occipital bone, and a sloping forehead. **c** T2-weighted sagittal image of an MRI scan showing a cerebral sickle, cerebellar tent, a defect of the cerebrum, a hypoplastic cerebellum, a normal brain stem, and excess cerebrospinal fluid

**Fig. 3:**
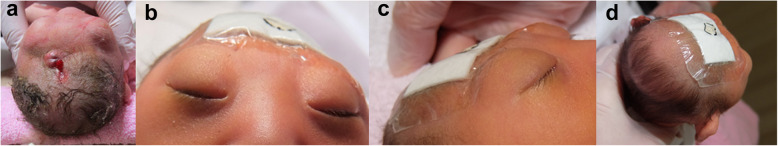
Images of the head and facial features of the infant after birth. **a** A skin defect without scalp rugae. **b** Front view of the face and **c** lateral view of the head. Both eyes and the nose appeared normal, but the head was flat above the forehead with a suspected partial head defect. **d** demonstrates the sloping forehead

**Fig. 4:**
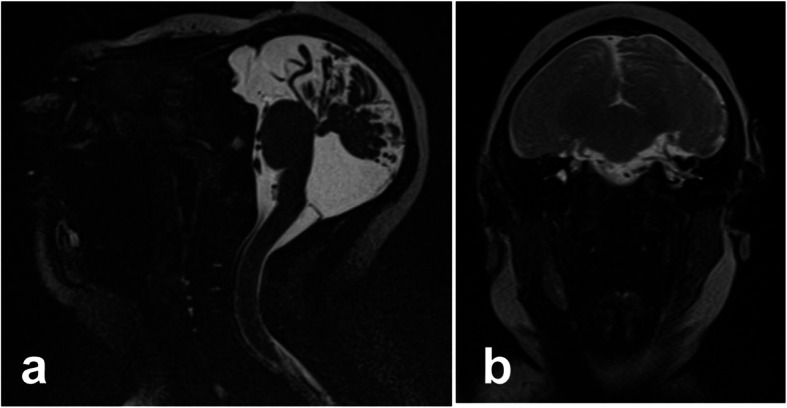
Magnetic resonance imaging (MRI) findings of the infant’s head. **a** T2-weighted sagittal image. **b** T2-weighted coronal image. On day 32 after birth, an MRI scan showed the absence of a cerebrum and a membranous structure in the region of the cerebrum with hydrocephalus

The patient was an 18-year-old woman, 2 gravida, nullipara. She had no remarkable medical or family history. She had a natural pregnancy and did not visit a hospital during the first trimester of pregnancy. The gestational age at the time of her initial hospital visit was estimated to be 15 weeks via measurement of the fetus on an ultrasound. Fetal biometry and estimated fetal weight were measured and evaluated according to the standardization committee of fetal measurement of the Japanese Society of Ultrasound in Medicine [7]. Head circumference (HC) was calculated from the diameter of biparietal diameter (BPD) and occipito-frontal diameter using the formula for an eclipse and evaluated according to the Hadlock et al. [8]. The BPD and femur length were 33 mm and 21 mm, respectively (Fig. 1a). At that time, there were no major anomalies, including the size and morphology of the fetal head. The estimated date of confinement was determined via ultrasound because of an unknown last menstrual period. At 23 weeks and 0 days of gestation, an ultrasound revealed a BPD of 40 mm (-5.3 standard deviations, SD), and the measured section of the BPD was poorly visualized. Additionally, observation of the fetal head using transvaginal ultrasound did not provide useful information about the cranial and cerebral defects. At 27 weeks and 5 days of gestation, an ultrasound revealed a BPD of 48 mm (-6.7 SD), however, there was difficulty visualizing the skull above the orbit and the overall growth, except for the head, was within the normal size range. At 29 weeks and 0 days of gestation, an ultrasound revealed a BPD of 43.6 mm (-8.9 SD) and a HC of 15.19 mm (-12.4 SD) (Fig. 1b and c). At 31 weeks and 6 days of gestation, we explained to the patient and the family that there was a possibility of microencephaly or anencephaly, and that anencephaly would result in a poor prognosis. We proposed magnetic resonance imaging (MRI) to the patient for a definitive diagnosis of the fetal head anomaly. However, the patient and the family refused further examination. At 34 weeks and 4 days of gestation, the patient was admitted for induction of labor due to the patient preferences. Elevation of the fetal head due to cervical dilatation by insertion of a metreurynter permitted confirmation of the structure of the fetal skull. MRI and 3-dimensional computed tomography (3D-CT) were performed to confirm the absence of anencephaly. 3D-CT revealed the presence of a fetal skull, a prominent occipital bone, and a sloping forehead (Fig. 2a and b). MRI showed a cerebral sickle, cerebellar tent, cerebral defects, a hypoplastic cerebellum, and a normal brain stem (Fig. 2c). The presence of the skull, cerebellum, and brainstem allowed us to diagnose the fetus with microhydranencephaly, rather than anencephaly. This diagnosis indicated that the fetus may be able to survive after delivery. Thus, the induction of labor was stopped, and the patient and the family were counseled for treatment. Vaginal delivery was scheduled with an exception for a cesarean section in the case of maternal indication. For the infant, noninvasive resuscitation treatment, except for tracheal intubation and chest compression, was scheduled for after delivery. Because the patient had cancelled many scheduled visits, she was hospitalized until delivery. At 36 weeks and 6 days of gestation, she had a premature rupture of the membranes, and delivered vaginally the following day without shoulder dystocia due to the small fetal head. The baby was a male weighing 2342 g, with a HC of 24 cm (-5.4 SD according to the Japanese neonatal data [9]) and Apgar scores of 1, 5, and 8 after 1, 5, and 10 min, respectively. Umbilical artery blood pH, pCO_2_, pO_2_, and base excess were 7.34, 42.7 mm Hg, 22.5 mm Hg, and − 3.5 mmol/L, respectively. After birth, the baby had spontaneous respiration. Both eyes and the nose had a normal appearance, but the head was flat above the forehead, with a suspected partial head defect with a sloping forehead (Fig. 3b and c, and 3d). Skin defects were found in the parietal region (Fig. 3a), and encephalocele was suspected. The skin lesion was taped, and healing was confirmed after 12 days, with no signs of infection or leakage of cerebrospinal fluid. Although the sucking reflex was observed, swallowing movements appeared difficult. On day 11 after birth, a gastric tube was inserted, and tube feeding was started. On day 32 after birth, an MRI revealed the absence of cerebral tissue; instead, a membranous structure was present and hydrocephalus was evident (Fig. 4), which was consistent with microhydranencephaly. Screenings for rubella virus, herpesvirus, cytomegalovirus, and toxoplasma infections causing hydrocephalus were all negative. Chromosomal analysis with G-banding yielded 46, XY, with and inv (9) (p12 q13) harboring less clinically significant abnormalities. The baby received desmopressin acetate due to central diabetes insipidus 6 months after birth.

## Discussion and Conclusions

We encountered a rare case of microhydranencephaly that was diagnosed prenatally. In this case, prenatal differentiation between microhydranencephaly and anencephaly or hydranencephaly was difficult with ultrasonography alone, and CT and MRI were required for a definitive diagnosis.

Microhydranencephaly is a malformation combining microcephaly and hydranencephaly. Although the etiology of microhydranencephaly remains elusive, several developmental processes have been proposed. Hydranencephaly is a malformation in which all or most of the cerebral hemispheres are absent, and are replaced by membranous structures filled with cerebrospinal fluid. Cerebral blood vessel circulation disturbances, such as bilateral carotid artery occlusion, can be drug-induced, iatrogenic, or caused by viral infection or genetic factors, and may occur in the first trimester of pregnancy, resulting in cerebral destruction in the area supplied by the anterior and middle cerebral arteries during the second or third trimester of pregnancy [5]. When brain tissue is destroyed or fails to develop, brain volume and intracranial pressure decrease, followed by the collapse and accumulation of the skull. Abnormal development of the skull interferes with the development of the entire head, resulting in microcephaly. This progression from hydranencephaly to microcephaly is called the fetal brain disruption sequence (FBDS) [1]. Russell et al. reported three cases of microhydranencephaly with skull overlapping, visible processes of the occipital bone, and wrinkles on the scalp, which corresponded to FBDS [1].

Similar cases of FBDS have been reported, and its characteristics have become clearer. Corona-Rivera et al. reviewed and reported 20 cases of FBDS, observing that the most common features of FBDS were microcephaly, a normal scalp, skull overlapping, and scalp wrinkles [4]. CT imaging revealed abnormal intracranial findings such as hydranencephaly, cerebral atrophy, ventricular enlargement, cerebellar atrophy, intracranial calcification, and intracranial hemorrhage [4]. Contrastingly, although marked microcephaly and hydranencephaly are common to FBDS, cases with normal cranial morphology have been reported [10]. Our patient had a scalp defect without apparent skull overlapping or scalp wrinkles, and the cerebrum was completely absent, with no cerebral atrophy. Taken together with previous reports, our case suggests that there may be phenotypic variations in FBDS.

FBDS is classified by morphological features of the skull and brain, but recent reports have revealed that certain genetic abnormalities are associated with the disease. Alexander et al. reported two cases of FBDS in sisters, suggesting a possible genetic cause [2]. Familial and sporadic cases of FBDS were reported and screened for via chromosomal mapping and genetic abnormalities. Mutations or deletions in neurodevelopment protein 1 (NDE1) have been reported in FBDS [3, 6, 10–13]. The NDE1 gene is located on chromosome 16p13.11, and encodes a protein with a role of mitosis, which is necessary for cerebral cortical development [14, 15]. In the present case, the only abnormality appeared to be the normal chromosomal subtype, and the examination for *NDE1* abnormality was not carried out. However, counseling before subsequent pregnancies should be considered due to the risk of familial microhydranencephaly.

Currently, 35 cases of microhydranencephaly or FBDS have been identified, including this report [1–4, 6, 10, 11, 16–24]. Among them, there were 15 cases of suspected or diagnosed microhydranencephaly or FBDS before birth (Table 1). The median gestational age at prenatal diagnosis was 28 weeks (range, 23–37 weeks). No cases showed microcephaly in the first trimester. Ultrasound was used for prenatal diagnosis in all cases, and MRI with ultrasound was used in four cases, whereas CT was used for prenatal diagnosis in the present case. An ultrasound examination confirmed fetal microcephaly in 14 cases. MRI and 3D-CT findings revealed microcephaly, hypoplasia or defects of the cerebrum, hypoplasia of the cerebellum, hydrocephalus, excess cerebrospinal fluid, a prominent occipital bone, and a sloping forehead in our patient. Ultrasonography can diagnose microcephaly, but a qualitative diagnosis of the skull and brain is difficult, and MRI or 3D-CT may be useful for a definitive prenatal diagnosis of microhydranencephaly.

**Table 1:** Published cases of prenatally diagnosed microhydranencephaly or fetal brain disruption sequence

First author (year)	GW at delivery	Delivery Method	Sex	Fetal birth weight (g)	OFC at birth (SD)	Overlapping suture	Prominent occipital bone	Sloping forehead	Scalp rugae	Normal scalp hair	Cerebral abnormality	Hydrocephalus	GW at prenatal diagnosis	Fetal findings at prenatal diagnosis	Imaging modalities used for prenatal diagnosis	Chromosomal abnormality	Prognosis
Russell (1984) [1]	37	NS	F	2400	-6.4	+	+	+	+	+	Severe atrophy	+	36	Microcephalus	US	Not tested	Death at 4 months
	Term	NS	F	1786	-7.5	+	+	+	+	+	Cerebrum defect	+	NA	Microcephalus	US	Not tested	Alive at 4 months
	Term	NS	M	1960	-6.6	+	+	NS	+	+	Atrophy and calcification	+	34	Microcephalus	US	Normal karyotype	Death at 23 days
Bönnemann (1990) [7]	35	VD	F	1670	-6.0	+	+	-	+	+	Atrophy	+	NA	Microcephalus	US	NS	NS
Alexander (1995) [2]	Term	VD	F	4200	-0.6	NS	NS	-	+	+	Severe atrophy	+	37	Microcephalus	US	Normal karyotype	Alive at 2.5 years
Gabis (1997) [19]	38	CS	M	NS	-0.9	+	NS	+	NS	+	Porencephaly	-	30	Microcephalus, intracranial hemorrhage, porencephalic cyst	US	NS	NS
DeJonge (1997) [20]	34	NS	F	2014	NS	+	+	+	+	+	Cerebrum defect	NS	28	Microcephalus	US	Normal karyotype	Death at 1 day
Villó (2001) [23]	38	VD	F	2055	-5.0	+	+	NS	+	+	Severe atrophy	+	25	Microcephalus	US	Normal karyotype	Alive at 3 years
Schram (2004) [3]	38	NS	M	2880	-2.8	+	+	+	+	+	Severe hypoplasia	+	28	Occipital flattening	US	Normal karyotype	Death at 2 years
	36	VD	M	2590	-3.4	+	NS	-	+	+	Aplasia and atrophy	+	36	Microcephalus, small brain	US	Normal karyotype	Death at 1 month
	33	VD	F	2050	-1.0	+	NS	-	+	+	Atrophy and calcification	+	26	Microcephalus, small brain, hypoplastic cerebellum, normal brain stem, hydrocephalus,excess amount of cerebrospinal fluid	US, MRI	Normal karyotype	Death at 1 day
Behunova (2010) [24]	40	NS	M	3770	-2.5	NS	NS	+	+	+	Severe atrophy	+	28–31	Microcephalus	US	Normal karyotype	Alive at 5.5 years
Abdel-Salam (2015) [6]	Term	CS	M	3000	-3.0	+	+	NS	NS	NS	Severe hypoplasia	+	28	Microcephalus, hypoplastic cerebrum, hypoplastic cerebellum, hypoplastic brain stem, hydrocephalus	US, MRI	NS	Death at several days
	28	VD	F	NS	NS	+	+	+	NS	NS	Severe hypoplasia	+	24	Microcephalus, hypoplastic cerebrum, hypoplastic cerebellum, hypoplastic brain stem, hydrocephalus, prominent occipital bone	US, MRI	Normal karyotype	Termination
Omoto (present case)	37	VD	M	2342	-5.4	-	+	+	-	Partial defect	Cerebrum defect	+	23	Microcephalus, cerebrum defect, cerebellum hypoplasia, normal brain stem, hydrocephalus, excess amount of cerebrospinal fluid, prominent occipital bone, sloping forehead	US, MRI, CT	46, XY, inv(9)(p12q13)	Alive at 18 months

*GW* Gestational week, *SD* Standard deviation, *OFC *Occipitofrontal circumference, *NS *Not stated, *VD *Vaginal delivery, *CS *Cesarean section, *M *male, *F *Female, *US *Ultrasonography, *MRI *Magnetic resonance imaging, *CT *Computed tomography

The prognosis in anencephaly is abysmal, often resulting in intrauterine fetal death before or during delivery. Survival at birth is impossible, and treatment is not available [25]. The prognosis of microhydranencephaly is also generally poor, although death does not occur immediately after birth. Of the 15 microhydranencephaly cases, including ours, diagnosed prenatally [1–3, 6, 17, 19, 20, 23, 24], 7 cases (47%) died over a period of 2 years after birth, and 6 cases died during the first 4 months of life, highlighting the poor prognosis.

Prenatal and differential diagnoses of microhydranencephaly and anencephaly are important for perinatal management because of differences in prognosis. Anencephaly is a serious developmental disorder of the central nervous system caused by a neural tube obstruction disorder. Anencephaly is characterized by a defect in the skull, as well as the brain parenchyma, which is a clear distinction from microhydranencephaly [26]. In this case, although the size of the head was normal in the first trimester, the head was smaller than average in the second trimester. Transvaginal ultrasound of the fetal head did not provide sufficient visualization of the cranium and brain. Therefore, differentiating between anencephaly and microhydranencephaly was difficult. The use of a balloon in the cervical canal enabled us to observe the fetal head in detail. However, this method is invasive and is problematic for diagnosis due to the potential of premature labor induction [27]. This observation suggests that raising the fetal head manually may be useful for its observation during the second trimester.

On the other hand, a differential diagnosis between microhydranencephaly and hydranencephaly is difficult. Hydranencephaly is characterized by marked cerebral hemispheric atrophy and hydrocephalus, with a variable head circumference ranging from normal to microcephaly or macrocephaly [12]. Hydranencephaly appears to stem from either a massive brain infarction from bilateral carotid artery occlusion or from primary agenesis of the neural wall [28, 29]. Microhydranencephaly and hydranencephaly may be overlapping diseases, and previously reported cases of hydranencephaly with microcephaly could have been diagnosed as microhydranencephaly.

In conclusion, we report a case of microhydranencephaly that was difficult to distinguish from anencephaly due to difficulty in observing the fetal head. Our report highlights that use of multiple imaging modalities and physical manipulation of the fetal head are required for accurate prenatal diagnosis. Considering that the prognosis of microhydranencephaly is quite different from that of anencephaly after birth, a prenatal diagnosis is crucial to prepare for perinatal management of the mother and baby.

## Data Availability

Not applicable.

## Abbreviations

BPD: Biparietal diameter
FBDS: Fetal brain disruption sequence
HC: Head circumference
MRI: Magnetic resonance imaging
SD: Standard deviation
3D-CT: 3-dimensional computed tomography

## References

[1] Russell LJ, Weaver DD, Bull MJ, Weinbaum M (1984). In utero brain destruction resulting in collapse of the fetal skull, microcephaly, scalp rugae, and neurologic impairment: the fetal brain disruption sequence. Am J Med Genet.

[2] Alexander IE, Tauro GP, Bankier A (1995). Fetal brain disruption sequence in sisters. Eur J Pediatr.

[3] Schram A, Kroes HY, Sollie K, Timmer B, Barth P, van Essen T (2004). Hereditary fetal brain degeneration resembling fetal brain disruption sequence in two sibships. Am J Med Genet A.

[4] Corona-Rivera JR, Corona-Rivera E, Romero-Velarde E, Hernandez-Rocha J, Bobadilla-Morales L, Corona-Rivera A (2001). Report and review of the fetal brain disruption sequence. Eur J Pediatr.

[5] Cecchetto G, Milanese L, Giordano R, Viero A, Suma V, Manara R (2013). Looking at the missing brain: hydranencephaly case series and literature review. Pediatr Neurol.

[6] Abdel-Salam GM, Abdel-Hamid MS, El-Khayat HA, Eid OM, Saba S, Farag MK (2015). Fetal brain disruption sequence versus fetal brain arrest: A distinct autosomal recessive developmental brain malformation phenotype. Am J Med Genet A.

[7] Shinozuka N (2011). Fetal biometry and fetal weight estimation: JSUM standardization. The Ultrasound Review of Obstetrics Gynecology.

[8] Hadlock FP, Deter RL, Harrist RB, Park SK (1984). Estimating fetal age: computer-assisted analysis of multiple fetal growth parameters. Radiology.

[9] Itabashi K, Miura F, Uehara R, Nakamura Y (2014). New Japanese neonatal anthropometric charts for gestational age at birth. Pediatr Int.

[10] Kavaslar GN, Onengut S, Derman O, Kaya A, Tolun A (2000). The novel genetic disorder microhydranencephaly maps to chromosome 16p13.3-12.1. Am J Hum Genet.

[11] Guven A, Gunduz A, Bozoglu TM, Yalcinkaya C, Tolun A (2012). Novel NDE1 homozygous mutation resulting in microhydranencephaly and not microlyssencephaly. Neurogenetics.

[12] Paciorkowski AR, Keppler-Noreuil K, Robinson L, Sullivan C, Sajan S, Christian SL (2013). Deletion 16p13.11 uncovers NDE1 mutations on the non-deleted homolog and extends the spectrum of severe microcephaly to include fetal brain disruption. Am J Med Genet A.

[13] Abdel-Hamid MS, El-Dessouky SH, Ateya MI, Gaafar HM, Abdel-Salam GMH (2019). Phenotypic spectrum of NDE1-related disorders: from microlissencephaly to microhydranencephaly. Am J Med Genet A.

[14] Alkuraya FS, Cai X, Emery C, Mochida GH, Al-Dosari MS, Felie JM (2011). Human mutations in NDE1 cause extreme microcephaly with lissencephaly [corrected]. Am J Hum Genet.

[15] Bakircioglu M, Carvalho OP, Khurshid M, Cox JJ, Tuysuz B, Barak T (2011). The essential role of centrosomal NDE1 in human cerebral cortex neurogenesis. Am J Hum Genet.

[16] Moore CA, Weaver DD, Bull MJ (1990). Fetal brain disruption sequence. J Pediatr.

[17] Bonnemann CG, Meinecke P (1990). Fetal brain disruption sequence: a milder variant. J Med Genet.

[18] Rasmussen S, Frias J (1990). Fetal brain disruption sequence: a brief case report. Dysmorphology clinical genetics.

[19] Gabis L, Gelman-Kohan Z, Mogilner M (1997). Microcephaly due to fetal brain disruption sequence. Case report. J Perinat Med.

[20] DeJonge M, Poulik J (1997). Pathological case of the month. Fetal brain disruption sequence. Arch Pediatr Adolesc Med.

[21] Gautam P, Phadke SR (2000). Fetal brain disruption sequence. Indian Pediatr.

[22] Bellini C, Massocco D, Serra G (2000). Prenatal cocaine exposure and the expanding spectrum of brain malformations. Arch Intern Med.

[23] Villo N, Beceiro J, Cebrero M, de Frias EG (2001). Fetal brain disruption sequence in a newborn infant with a history of cordocentesis at 21 weeks gestation. Arch Dis Child Fetal Neonatal Ed.

[24] Behunova J, Zavadilikova E, Bozoglu TM, Gunduz A, Tolun A, Yalcinkaya C (2010). Familial microhydranencephaly, a family that does not map to 16p13.13-p12.2: relationship with hereditary fetal brain degeneration and fetal brain disruption sequence. Clin Dysmorphol.

[25] Obeidi N, Russell N, Higgins JR, O’Donoghue K (2010). The natural history of anencephaly. Prenat Diagn.

[26] Padmanabhan R (2006). Etiology, pathogenesis and prevention of neural tube defects. Congenit Anom (Kyoto).

[27] Greenberg V, Khalifeh A (2015). Intracervical Foley balloon catheter for cervical ripening and labor induction: A review. Semin Perinatol.

[28] Pretorius DH, Russ PD, Rumack CM, Manco-Johnson ML (1986). Diagnosis of brain neuropathology in utero. Neuroradiology.

[29] Greene MF, Benacerraf B, Crawford JM (1985). Hydranencephaly: US appearance during in utero evolution. Radiology.

